# Enzymatic modulation of hulless barley starch: Unraveling structural origins of altered digestibility and functional properties

**DOI:** 10.1016/j.fochx.2026.104169

**Published:** 2026-07-07

**Authors:** Xinzhe Gu, Zufei Cao, Yu Wang, Mingyuan Zheng, Xiaohui Liu, Wei Zhang

**Affiliations:** a College of Food Science and Technology, Yunnan Agriculture University, Kunming, 650500, People's Republic of China; b Department of Food Science and Technology, School of Agriculture and Biology, Shanghai Jiao Tong University, Shanghai, 200240, People's Republic of China; c College of Tea Science, Yunnan Agricultural University, Kunming 650500, People's Republic of China; d College of Food Science and Technology, Nanjing Agricultural University, Nanjing 210095, People's Republic of China

**Keywords:** Hulless barley starch, Pullulanase, Amyloglucosidase, Structure, *In vitro* digestibility

## Abstract

This study investigated the effects of pullulanase and amyloglucosidase modification on the *in vitro* digestibility, multi-scale structure, and processing characteristics of hulless barley starch. Both enzymatic treatments reduced the rapidly digestible starch (RDS) content and increased the total content of slowly digestible starch (SDS) and resistant starch (RS). Pullulanase showed superior performance to amyloglucosidase. Treatment at 60 °C enhanced anti-digestibility, with the B-pullulanase-50 sample achieving the highest SDS + RS content (31.95%). Amylose content increased with enzyme concentration. XRD analysis showed a transition from A-type to B-type crystal structure. FTIR and Raman spectroscopy revealed enhanced short-range order and increased helical structures. Particle size and SEM results indicated granule aggregation and morphological changes, including surface erosion and swelling. DSC analysis showed elevated gelatinization temperatures. Rheological tests demonstrated pseudoplastic behavior with reduced consistency and enhanced elastic character post-modification. These results suggested enzyme modification can effectively tailor the digestibility and physicochemical properties of hulless barley starch.

## 1 Introduction

Hulless barley (*Hordeum vulgare L.var.nudum hook. f*) serves as the predominant food crop in the Qinghai-Tibet Plateau region and is the staple food for ethnic groups such as the Tibetans (Bai et al., 2023). As an ancient crop endemic to this area, it has been cultivated for over 3500 years. Hulless barley plays a pivotal role in maintaining the ecological balance of the plateau (Pan et al., 2023). Its well-developed root system and strong resilience to cold and drought not only reinforce its function as a ‘food security barrier on the plateau’ but also highlight its importance as an ecological crop for soil and water conservation (Fu et al., 2025). The irreplaceable geographical and cultural significance of hulless barley renders it an invaluable model for studying the adaptability of organisms to extreme environments in the Qinghai-Tibet Plateau (Luo et al., 2025). Additionally, the unique nutritional profile of hulless barley, characterized by high protein, high fiber, high vitamins, low fat and low sugar, along with its rich content of functional bioactive components such as β-glucan and phenolic compounds, has positioned it as a significant research focus within the healthy food domain (Cao et al., 2025; Ge et al., 2025).

However, starch is the predominant component in hulless barley. Currently, the global incidence of chronic diseases such as type 2 diabetes, cardiovascular diseases and obesity is increasing annually (Janssen, 2024; Mrozovski, 2025). One of the key contributing factors is the excessive and prolonged consumption of starch-based foods (Albers et al., 2014; Çavuşoğlu et al., 2025). Therefore, there is growing interest in controlling the digestibility of starch in food and elucidating the factors and mechanisms that influence starch digestion (Li et al., 2020b). On the basis of preserving the inherent nutritional characteristics of hulless barley products, enhancing the resistance to digestion of hulless barley starch can contribute to improving its functional food value.

The starch in hulless barley exhibits distinct functional components and structural characteristics compared to common starches such as corn, wheat, and potato starch (Chen et al., 2023). The proportion of resistant starch ranges from 10% to 15%, which not only functions as a prebiotic to promote intestinal health but also makes it suitable for the development of low-GI foods (Li et al., 2024). Furthermore, hulless barley starch is characterized by small particle size, high crystallinity and a low gelatinization temperature (Pan et al., 2024a). These properties make it an ideal candidate for use as a thickener and stabilizer in food processing applications (Pan et al., 2024b), and are subject to modification by processing conditions (Zhang et al., 2025a). It also contains antioxidant components and exhibits potential bioactivities, such as anti-inflammatory effects. A recent study by Yang et al. (2026) on the bioactive polysaccharides from fresh *Rehmannia radix* systematically elucidated their structure-activity relationship, revealing that multi-scale structural features critically determine their rheological and biological functions. This provides a valuable methodological and conceptual reference for investigating the functional attributes of macromolecules in hulless barley. However, when compared with staple grain starches such as wheat and corn, research on hulless barley starch remains in its infancy. Within the existing literature, in-depth investigations into its molecular structure, processing properties and functional activities are limited. Furthermore, its application in industrial domains, including pharmaceutical carriers and biodegradable materials, remains largely unexplored.

Starch is composed of two types of glucose polymers: amylose, a linear polysaccharide formed by α-d-glucopyranose units linked through α-(1 → 4) glycosidic bonds. And amylopectin, a highly branched polymer of α-d-glucopyranose units with α-(1 → 4) linkages in the main chains and α-(1 → 6) linkages at branch points. According to prior research, the fine structural features of starch play a critical role in determining its functional properties, such as digestibility, gelatinization behavior and rheological characteristics (Guo et al., 2024; Ji et al., 2021). More specifically, starch digestibility is influenced by the amylose-to-amylopectin ratio, the relative proportions of crystalline and amorphous regions, and the presence or absence of double helical structures within the starch granules (Wang et al., 2023). These factors can be modulated through physical, chemical and enzymatic treatments. Studies have demonstrated that enzymatic modification effectively reduces the digestibility of starch while increasing the contents of RS and SDS (Sidhu et al., 2025). Moreover, enzymatic approaches offer distinct advantages, including mild reaction conditions, high specificity and high product purity, making them a promising strategy for tailoring starch functionality.

During the hydrolysis of starch side chains, pullulanase is the most commonly employed enzyme. As a specific amylase, pullulanase selectively cleaves the α-1,6 glycosidic bonds at the branching points of amylopectin, excising entire branch structures and generating amylose (Chi et al., 2023). To date, numerous studies have reported the application of pullulanase in reducing starch digestibility. For instance, Semwal and Meera (2023) demonstrated that the combined treatment of pullulanase and infrared radiation increased the amylose content and crystallinity of sorghum starch, thereby effectively reducing its digestibility. Liu et al. (2023) further explored the potential of pullulanase in resistant starch production. And they found that pullulanase was more efficient than other reported debranching enzymes in producing resistant starch from peas. Additionally, amyloglucosidase specifically hydrolyzes α-1,4 glycosidic bonds from the non-reducing ends of starch to produce glucose (Warren et al.,2015). Das et al. (2022) applied amyloglucosidase to green banana powder, significantly increasing the content of resistant starch.

However, its application remains limited in current research on resistant starch production. Critically, despite the proven efficacy of pullulanase and amyloglucosidase in modifying starches from sources like sorghum, pea and banana, systematic investigations into their effects on hulless barley starch are conspicuously absent, either individually or synergistically. Hulless barley starch possesses a unique combination of structural features, including small granule size, high crystallinity and a distinct amylose/amylopectin architecture, which differs fundamentally from the previously studied starches. This structural uniqueness implies that the enzymatic hydrolysis pattern, and consequently the resulting changes in multi-scale structure and digestibility, cannot be reliably predicted from existing literature. In particular, no studies have elucidated how these enzymes alter the hierarchical structure (from molecular to crystalline to granular levels) of hulless barley starch to control its digestive fate. The lack of such studies hinders the rational design of enzymatic processes to tailor the digestibility of foods based on hulless barley.

Therefore, the first objective of our work was to investigate the effects of pullulanase and amyloglucosidase modification under varying treatment concentrations and temperatures on the digestive performance of hulless barley starch. The second objective was to analyze the intrinsic correlations between the digestibility of hulless barley starch and the crystallinity, short-range and long-range ordered structures, particle size distribution and particle morphology of starch, and to elucidate the molecular mechanism underlying how changes in starch structure affect its digestive performance. These outcomes are of great significance for the development of starch-based foods with tailored digestibility.

## 2 Materials and methods

### 2.1 Starch extraction

The hulless barley was procured from Tibet Zangke Food Co., Ltd., China. A 300 g of grains were soaked overnight in 1 L of deionized water. After the soaking period, the grains were drained and mixed with 1 L of a 0.2% NaOH solution. The resultant mixture was pulped and filtered through a 200-mesh sieve, with the filtrate being collected. The material retained on the sieve was subsequently dissolved in 500 mL of a 0.2% NaOH solution. Following thorough stirring, the mixture was filtered again through a 200-mesh sieve. This process was repeated three times, and the final filtrate was collected for further use.

The combined filtrates were stirred overnight for extraction and subsequently neutralized with dilute hydrochloric acid to adjust the pH to 7.0. The mixture was then centrifuged at 10,000 ×*g* (*g* = 9.8 m·s^−2^) for 10 min at 4 °C (TGL-18 M, Shanghai Luxiangyi Centrifuge Instruments Co., Ltd., China). Following the removal of the supernatant, as well as the yellow-brown substances in the upper layer and impurities at the bottom, the remaining white precipitate was dispersed in 500 mL of deionized water. The resulting suspension was stirred for 60 min and subsequently centrifuged. This procedure was repeated three times. Finally, the resultant white wet precipitate was freeze-dried using a vacuum freeze dryer (CTFD-18 T, Qingdao Yonghe Chuangxin Electronic Technology Co., Ltd., China), manually ground into a fine powder for 5 min using an agate mortar and pestle, sieved through a 200-mesh sieve, and stored in a dry environment at room temperature.

### 2.2 Modification of hulless barley starch

#### 2.2.1 Pullulanase

Two grams of each of the three hulless barley starch samples were weighed, and each sample was dispersed in 30 mL of Tris-HCl (50 mM, pH 5.0) buffer solution. Add 10 U, 40 U and 100 U of pullulanase (Promozyme D2, activity 1350 NPUN/g, Shanghai Aladdin Biochemical Technology Co., Ltd.) to the respective samples, thereby adjusting the enzyme concentration to 5 U/g, 20 U/g and 50 U/g. The reaction mixtures were placed in a water bath shaker (SHZ-82, Changzhou Jintan Hengfeng Instrument Manufacturing Co., Ltd., China) at 50 °C and 200 rpm/min for 12 h. After enzymatic hydrolysis, the mixtures were centrifuged at 1500 ×*g* for 10 min. The precipitates were dispersed in 10 mL of 75% ethanol for enzyme inactivation, followed by three washes with distilled water. The samples were then freeze-dried, ground, and sieved through a 200-mesh sieve. In this study, the prefix A denoted hydrolysis at 50 °C and B denoted hydrolysis at 60 °C. The modified starch samples obtained at 50 °C were designated A-pullulanase-5, A-pullulanase-20, and A-pullulanase-50, which were subsequently stored at room temperature.

Another three samples of hulless barley starch were taken and the mixed reaction system was prepared according to the above operation. Enzymatic hydrolysis was carried out at 60 °C for 12 h to obtain B-pullulanase-5, B-pullulanase-20 and B-pullulanase-50.

#### 2.2.2 Amyloglucosidase

In accordance with the method for modifying hulless barley starch using pullulanase, the starch samples were enzymatically hydrolyzed with amyloglucosidase (A7420, activity 30–60 U/mg, Sigma-Aldrich) and subsequently designated as A-AMG-5, A-AMG-20, A-AMG-50, B-AMG-5, B-AMG-20, and B-AMG-50.

### 2.3 Determination of *in vitro* digestibility

The determination of *in vitro* digestibility of hulless barley starch was carried out according to the method by Wang (2017). Three grams of porcine pancreatic α-amylase was weighed and dissolved in 20 mL of distilled water, and the mixture was stirred for 10 min. Subsequently, the solution was centrifuged at 4 °C at 1500 ×*g* for 30 min. And 13.5 mL of the supernatant was transferred to a beaker, then 0.7 mL of amyloglucosidase and 0.8 mL of distilled water were added, ensuring thorough mixing for future use.

A hundred milligrams of hulless barley starch were weighed and 2 mL of acetate buffer (0.1 M, pH 5.2) was added. The mixture was heated to boiling and the boiling temperature was maintained for 30 min. After cooling the mixture to 37 °C, 0.5 mL of the previously prepared mixed enzyme solution was added. Subsequently, the hydrolysis reaction was conducted in a shaking water bath at 37 °C with a speed of 180 rpm/min. At 20 and 120 min into the reaction, 0.1 mL of the hydrolysate was withdrawn and added to 5 mL of a 75% ethanol solution to terminate the reaction. The mixture was then centrifuged at 1500 ×*g* for 10 min to collect the supernatant. The glucose concentration in the hydrolysate was determined using the GOPOD method, and the quantities of the RDS, SDS and RS contents were calculated.

The percentages of RDS, SDS and RS were determined using the following formula:

$$\mathrm{RDS}=\frac{G_{20}\times 0.9}{\mathrm{TS}}\times 100\%$$

$$\mathrm{SDS}=\frac{\left(G_{120}-G_{20}\right)\times 0.9}{\mathrm{TS}}\times 100\%$$

RS=100−RDS−SDS

Where the glucose content produced by starch hydrolysis for 20 min and 120 min was expressed as *G*_20_ and *G*_120_, respectively, and TS was starch mass before digestion.

### 2.4 Determination of amylose content

The amylose content of the hulless barley starch was determined using the methodology outlined by Shi et al. (2023). One hundred milligrams of defatted starch was placed in a 50 mL centrifuge tube, and 1 mL of anhydrous ethanol was added to moisten the sample, followed by the addition of 9 mL of 1 M NaOH solution. The mixture was thoroughly mixed and then heated in a boiling water bath for 10 min. After cooling to room temperature, 5 mL of this mixture was transferred to a 100 mL volumetric flask. Then 0.5 mL of 1 M acetic acid solution was added, followed by the addition of 1 mL of iodine reagent. The solution was diluted to the mark with distilled water, allowed to stand for 20 min for color development, and the absorbance was measured at 620 nm. A standard curve was constructed using potato amylose and corn amylopectin, allowing for the calculation of amylose content in hulless barley starch based on this curve.

### 2.5 Determination of swelling power and solubility

A total of 100 mg of hulless barley starch was added to 10 mL of distilled water, mixed thoroughly, and heated in a water bath at 85 °C for 30 min with continuous stirring. After cooling to room temperature, the mixture was centrifuged at 1500 ×*g* for 20 min. The precipitate and supernatant were collected separately, with the mass of the precipitate being meticulously recorded. Subsequently, the supernatant was subjected to freeze-drying, and the mass of the resultant residue was accurately measured.

The formula for calculating the swelling power of starch is as follows:

$$\text{Swelling power}=\frac{\text{Mass of the precipitate}}{\text{Mass of the starch}}$$

The formula for calculating the solubility of starch is as follows:

$\text{Solubility}=\frac{\text{Mass of residual residue}}{\text{Mass of starch}}$**×**100.

### 2.6 X-ray diffraction (XRD)

A multi-functional X-ray diffractometer (D8 ADVANCE Da Vinci, Bruker, Germany) was employed to analyze the crystalline structure of hulless barley starch. Powder samples were placed into a circular mold, compacted firmly, and subsequently transferred to the detection chamber. The test conditions were as follows: Cu Kα radiation with a wavelength of 1.542 Å, a tube voltage of 40 kV, and a tube current of 20 mA. Scanning was conducted over the range of 3–40° (2θ) with a step size of 0.02°. The XRD patterns of the samples were processed using MDI Jade 6 software, and the relative crystallinity was calculated based on the peak deconvolution method.

### 2.7 Fourier transform infrared spectroscopy (FTIR)

The infrared spectral characteristics of hulless barley starch were measured using a Fourier transform infrared spectrometer (Nicolet 6700, Thermo Fisher Scientific, USA). The sample powder was uniformly mixed with potassium bromide at a ratio of 1:100 and subsequently ground. A specific amount of the mixture was pressed into tablets and placed in the detection chamber for analysis. The test conditions were set as follows: a resolution of 4 cm^−1^, a scanning wavenumber range from 4000 to 400 cm^−1^, and air used as the background reference. The infrared spectrum of the sample was obtained by averaging the data from 16 consecutive scans. The infrared spectrum of the sample was obtained by averaging the data from 16 consecutive scans, and processed using Fourier self-deconvolution *via* OMNIC 8.2 software, followed by peak deconvolution. The peak areas at 1045, 1022, and 995 cm^−1^ were calculated to determine the intensity ratios R_1045/995_ and R_1022/995_.

### 2.8 Raman spectrum

The Raman spectral data of hulless barley starch were collected using a dispersive confocal Raman spectrometer (Senterra R200-L, Bruker, Germany). Before sample analysis, the instrument was calibrated for wavelength accuracy, and the background spectrum was recorded. Approximately 100 mg of the powdered sample was evenly distributed on a clean glass slide and gently flattened to ensure uniformity. Spectral scanning was conducted at an optimal position with the following parameters: a wavenumber range of 4400–200 cm^−1^, an excitation wavelength of 532 nm, and a laser power of 5 mW. Subsequently, the full width at half maximum (FWHM) of the characteristic band at 480, 855 and 1339 cm^−1^ was determined using Origin 9.1 software.

### 2.9 Determination of particle size and diameter distribution

The particle size analysis was performed using the Mastersizer2000 (S3500SI, Microtrac Inc., USA). A small quantity of hulless barley starch was prepared into a solution of a concentration of 1% (*w*/w). Following thorough mixing, starch slurry was transferred to the dispersion tank of a laser diffraction instrument. The refractive indices of starch and water were respectively configured to 1.533 and 1.33 at room temperature. Subsequently, the volumetric size distribution of the starch granules was recorded. The distribution patterns were automatically calculated using the integrated software of the laser diffraction particle size analyzer.

### 2.10 Scanning electron microscopy (SEM) analysis

The morphological changes of the starch samples were monitored using the high-throughput scanning electron microscope (Navigator-100, PolyBeam Technology, China). The starch to be tested was evenly distributed onto the sample stage equipped with conductive double-sided tape, and the stage was gently tapped to remove any loosely adhered powder particles. Subsequently, the sample stage was placed into the ion sputtering coater for gold deposition treatment. Finally, the samples were positioned inside the microscopic observation chamber, where the morphology of the starch was examined under magnifications of 3000×, 5000× and 10,000×, respectively. Micrographs were captured at an acceleration voltage of 2 kV.

### 2.11 Differential scanning calorimetry (DSC)

The thermodynamic properties of hulless barley starch were analyzed using a differential scanning calorimeter (DSC 204 F1, NETZSCH, Germany). Specifically, 3 mg of sample was placed in the high-pressure sample pan and mixed with 10 μL of deionized water. The sample aluminum pan was gently shaken to ensure uniform distribution of the mixture across the pan surface before sealing. Subsequently, the sealed pan was inserted into the DSC instrument for testing. The temperature program was set as follows: starting at 30 °C, the temperature was increased to 90 °C at a constant rate of 5 °C/min. Based on the resulting DSC cur*v*e, the onset phase transition temperature (*To*), peak temperature (*Tp*), end temperature (*Tc*), and gelatinization enthalpy (*ΔH*) of the starch samples were determined.

### 2.12 Rheological analysis

The rheological properties of hulless barley starch were characterized using a DHR-1 rheometer (TA Instruments, USA).

#### 2.12.1 Steady flow properties

A 5% (*w*/*v*) starch slurry was prepared and subjected to gelatinization in a water bath at 90 °C for 30 min with continuous stirring. After gelatinization, the sample was cooled to 25 °C. Rheological measurements were conducted using a parallel-plate system with a diameter of 40 mm. The gap was set to 1000 μm, which is much larger than the typical size of swollen granules, to minimize wall-slip artifacts. The measurement temperature was set at 25 °C, and the scanning strain was fixed at 1%. Prior to testing, the sample was equilibrated at 25 °C for 5 min to ensure thermal stability. Subsequently, the shear rate was increased logarithmically from 0.1 s^−1^ to 1000 s^−1^, followed by a decrease back to 0.1 s^−1^, to capture the steady flow cur*v*e and any thixotropic hysteresis.

The resulting data were analyzed using the power law equation:

σ = K·γ^n^.

where σ represents the shear stress (in Pascal, Pa), K represents the consistency coefficient (in Pa·s^n^), γ represents the shear rate (in inverse seconds, s^−1^), and n represents the dimensionless flow behavior index.

#### 2.12.2 Dynamic oscillatory properties

A 20% (*w*/*v*) starch slurry was transferred to a water-bath and heated at 95 °C for 30 min to fully gelatinize the starch, forming a homogeneous starch paste. Rheological measurements were conducted on a rheometer equipped with a parallel-plate measurement system. The plates had a diameter of 40 mm, and the gap between them was set at 1000 μm. The temperature-control program was precisely designed as follows: the sample was first equilibrated at 25 °C for 2 min to ensure thermal stability. Then, the temperature was increased from 25 °C to 90 °C at a constant rate of 2 °C/min, followed by a subsequent decrease back to 25 °C at the same rate of 2 °C/min. During the entire measurement, an oscillation amplitude of 2% and a frequency of 1 Hz were maintained. The dynamic changes in storage modulus (G'), loss modulus (G"), and loss tangent (tan δ = G”/G') were continuously monitored and recorded.

### 2.13 Statistical analysis

Each experiment was conducted as triplicate and statistical difference between treatments was determined by using variance analysis (one-way ANOVA) and Duncan's multiple comparison test (*p* < 0.05) using IBM SPSS Statistics software (Version 20, IBM Corp., USA). The tables contained the data having the mean and standard deviation of triplicated experiment results.

## 3 Results and discussions

### 3.1 *In vitro* digestibility

As shown in Table 1, native starch contained 90.32% RDS, 6.05% SDS, and 3.63% RS. In this study, enzymes were used to modify starch samples at temperatures below or above their gelatinization point (50 °C or 60 °C) to alter the RDS, SDS and RS contents.

**Table 1:** The effects of enzyme treatment on *in vitro* digestibility and physicochemical properties of hulless barley starch.

	**RDS/%**	**SDS/%**	**RS/%**	**Amylose**	**Swelling capacity** **(g/g)**	**Solubility (g/100 g)**
**Native starch**	90.32 ± 0.73a	6.05 ± 0.21f	3.63 ± 0.57j	22.61 ± 0.13 g	14.19 ± 0.28c	7.39 ± 0.58f
**A-pullulanase-5**	84.52 ± 0.81c	10.75 ± 0.63b	4.73 ± 0.92i	26.01 ± 0.18e	14.80 ± 0.11bc	8.79 ± 0.34e
**A-pullulanase-20**	81.12 ± 1.35de	11.96 ± 0.35b	6.92 ± 1.11 h	28.78 ± 0.05c	15.77 ± 0.14b	9.65 ± 1.22de
**A-pullulanase-50**	75.31 ± 0.59 g	13.48 ± 0.76a	11.21 ± 0.29d	29.50 ± 0.09bc	15.54 ± 0.25b	8.46 ± 0.85e
**B-pullulanase-5**	78.81 ± 2.13f	12.17 ± 1.44ab	9.02 ± 1.07f	27.93 ± 0.06d	12.32 ± 0.51d	10.32 ± 1.53d
**B-pullulanase-20**	71.80 ± 3.48 h	13.56 ± 1.82a	14.64 ± 2.12b	30.04 ± 0.12b	11.85 ± 0.14de	12.74 ± 1.66b
**B-pullulanase-50**	68.05 ± 2.93i	11.39 ± 2.13b	20.56 ± 1.45a	31.22 ± 0.05a	11.62 ± 0.37e	13.86 ± 2.01a
**A-AMG-5**	86.60 ± 1.44b	8.48 ± 0.47d	4.92 ± 1.00i	23.13 ± 0.18 g	16.39 ± 0.53b	9.76 ± 0.74de
**A-AMG-20**	82.89 ± 0.92d	9.46 ± 0.83c	7.65 ± 0.77gh	25.79 ± 0.11e	17.46 ± 0.61a	9.87 ± 1.29de
**A-AMG-50**	84.55 ± 1.13c	7.17 ± 0.24e	8.28 ± 0.80 g	28.66 ± 0.02c	17.94 ± 0.16a	8.14 ± 1.35f
**B-AMG-5**	80.71 ± 1.06e	8.83 ± 1.29 cd	10.46 ± 1.15e	23.84 ± 0.07f	13.08 ± 0.97d	11.83 ± 2.34c
**B-AMG-20**	78.66 ± 2.45f	8.42 ± 0.56d	12.92 ± 1.33c	25.53 ± 0.16e	12.84 ± 1.02d	10.46 ± 1.28d
**B-AMG-50**	75.51 ± 2.36 g	9.16 ± 1.41c	15.33 ± 1.59b	24.10 ± 0.28f	12.43 ± 0.68d	10.17 ± 1.11d

Data presented as Mean ± SD of three replicate values. Duncan's multiple comparison test (*p* < 0.05) of one-way ANOVA was performed to determine the differences in data. Different letters in the table indicated significant differences in values.

Starch samples treated with pullulanase exhibited significantly enhanced resistance to digestion compared with native starch. At 50 °C, the RDS content in treated samples ranged from 75.31% to 84.52%, SDS from 10.75% to 13.48%, and RS from 4.73% to 11.21%. Increasing pullulanase levels resulted in decreased RDS and increased SDS and RS contents. For instance, the SDS + RS content of the A-pullulanase-50 sample reached approximately 15%, higher than in native starch. At 60 °C, the SDS + RS content continued to increase with pullulanase dosage, with B-pullulanase-50 showing the highest value at 31.95%.

Amyloglucosidase also proved effective in regulating hulless barley starch digestibility. In samples A-AMG-5 through B-AMG-50, RDS ranged from 75.51% to 86.60%, SDS from 7.17% to 9.46%, and RS from 4.92% to 15.33%. Notably, at 60 °C, SDS + RS contents were higher than at 50 °C, and increasing enzyme levels further reduced RDS while boosting the RS content. The highest SDS + RS contents were observed in A-AMG-50 and B-AMG-50 at 15.45% and 24.49%, respectively.

Pullulanase specifically cleaves α-1,6 glycosidic bonds at amylopectin branch points, converting them into linear amylose (Chi et al., 2023). Amyloglucosidase hydrolyzes α-1,4 glycosidic bonds from the non-reducing end, releasing glucose (Warren et al., 2015). In this study, both enzymes hydrolyzed hulless barley starch into short-chain molecules. It was hypothesized that these short chains might rearrange into amorphous regions, single and double helices, and microcrystalline structures, consistent with the molecular rearrangement patterns observed in similar enzymatic hydrolysis systems (Ai & Jane, 2018). Regarding retrogradation, it was postulated that these short-chain molecules could tend to recrystallize into dense, helical structures *via* hydrogen bonding, potentially forming tightly packed starch crystals, as supported by the tendency of short starch chains, though direct experimental evidence for such retrogradation was not obtained in the present study.

Biocatalytic starch modification to control digestibility and nutritional properties has gained significant research interest. Studies have demonstrated the potential of these methods. For example, Hong et al. (2021) enhanced frozen noodle texture and cooking properties by adding pullulanase. Tu et al. (2021) found that pullulanase, combined with freeze-thaw cycles, increased free amylose in lotus seed starch, promoting starch-lipid (glycerol monostearate) complex formation and resistant starch content. Semwal and Meera (2023) used pullulanase and infrared radiation treatment on sorghum grains to raise amylose content and starch crystallinity, thus reducing starch digestibility. Pullulanase debranching is an efficient method for producing customized starch for specific dietary needs. Additionally, Dura et al. (2014) used α-amylase and amyloglucosidase to create porous corn starch, confirming that enzymatic modification results in aggregated, porous structures. Das et al. (2014) applied amyloglucosidase to green banana powder, increasing resistant starch content from 385 to 806 g/kg. However, research on biocatalytic modification of hulless barley starch was limited. This study demonstrated the strong potential of pullulanase and amyloglucosidase in regulating its digestibility. Further investigation into their effects on SDS/RS generation and starch structure is essential for industrial applications.

### 3.2 Amylose content, swelling capacity, and solubility

Table 1 presented the amylose content, swelling power and solubility of native starch and modified starch samples by pullulanase and amyloglucosidase. The native starch showed an amylose content of 22.61%. Irrespective of the hydrolysis temperature, modified starch samples by pullulanase demonstrated increased amylose content. For instance, the amylose contents after A-pullulanase-5, A-pullulanase-20, and A-pullulanase-50 treatments were 26.01%, 28.78%, and 29.50%, respectively; for B-pullulanase treatments, the corresponding values were 27.93%, 30.04%, and 31.22%, respectively (Table 1). The rise in pullulanase dosage led to a continuous increase in the amylose content, as pullulanase could specifically cleave α-1,6-glycosidic bonds in amylopectin to form amylose (Chi et al., 2023). A similar pattern was observed in the modified samples by amyloglucosidase. At 50 °C, the amylose content ranged from 23.13 to 28.66%; at 60 °C, it ranged from 23.84 to 25.53% (Table 1), both of which showed higher content than in the original starch. Modification with either of the enzymes significantly boosted the amylose content.

According to previous studies, the swelling force and solubility were related to the different proportions of amylose and amylopectin in starch and the differences in the starch structure (Dereje, 2021). When the temperature was lower than the enzymatic hydrolysis temperature, after the action of enzymes on starch samples, both the swelling force and solubility of the starch were enhanced. The ranges of the swelling force of starch modified by pullulanase or amyloglucosidase were 14.80–15.54 and 16.39–17.94 g/g, respectively, while the solubility ranges were 8.46–9.65 and 8.14–9.87 g/100 g, respectively (Table 1). The enzymatic hydrolysis by the enzymes enabled the amylopectin content in the starch to be hydrolyzed into more numbers of short-chain molecules. These short-chain molecules then reorient, coil, and align, which increases the amylose content in the starch system and accelerated the release of linear amylose from the starch as well as the entry of water molecules into the starch granules to cause their expansion (Wang et al., 2023).

At high hydrolysis temperatures, the solubilities of modified starches increased. Pullulanase- modified and amyloglucosidase-modified samples showed solubilities of 10.32–13.86 g/100 g and 10.17–11.83 g/100 g, respectively (Table 1). Notably, the swelling capacity of starch after enzymatic hydrolysis has decreased. The values of 11.62–12.32 and 12.43–13.08 g/100 g were obtained for pullulanase and amyloglucosidase, respectively (Table 1). These values may be attributed to the significant structural changes that occurred in the structure of starch after enzymatic hydrolysis at temperatures above the gelatinization temperature.

### 3.3 XRD analysis of starch

**Fig. 1:**
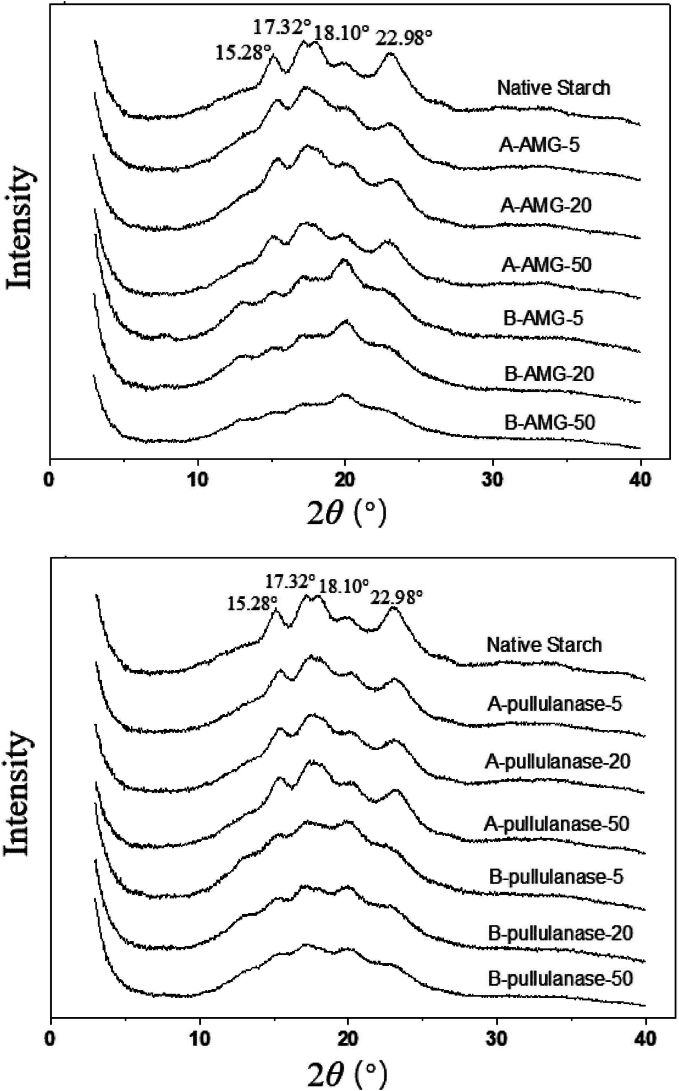
The variation in the XRD Diffractogram of starch samples modified by pullulanase and amyloglucosidase under different temperature and concentration conditions.

As presented in Fig. 1, native starch displayed distinct diffraction peaks at 2*θ* values of 15.28°, 17.32°, 18.10°, and 22.98°, indicating a typical A-type crystal structure and the presence of a sharp peak at approximately 2*θ* = 20° revealed *V*-type crystallization characteristics (Castillo-Paz et al., 2024).

From Fig. 1, after pullulanase treatment at 50 °C, the samples continued to demonstrate strong diffraction peaks at 15°, 20° and 23°, albeit the double-peaks at 17.32° and 18.10° had disappeared, implying a transformation of the A-type crystal structure. When the reaction temperature rose to 60 °C, characteristic peaks emerged at 5.73°, 12.87°, 17.02° and 23.88°, representing a typical B-type structure (Fig. 1) (Castillo-Paz et al., 2024). Similar results have also been reported by previous studies. For example, Bai et al. (2023) treated starch with ultrasound and heat combined with pullulanase. The results indicated that the starch modified by pullulanase changed from type A crystals to type B crystals. Liu et al. (2023) reported that natural pea starch showed a C-type crystal structure, and after modification with pullulanase, it transformed into a B-type crystal structure. In this study, because of the enzymatic hydrolysis effect of pullulanase, the amylopectin in starch was enzymatically hydrolyzed into more numbers of short-chain molecules. During the aging and re-crystallization processes of starch, these short-chain molecules formed closely packed double helix structures and aggregated into a complex B-type crystal structure through hydrogen bonding (Wang et al., 2023).

Starch treated with amyloglucosidase at 50 °C displayed strong diffraction peaks at approximately 15°, 17°, 20° and 23° (Fig. 1). The original double peaks were replaced by a more intense peak at approximately 17.0°, which may be linked to molecular rearrangement. When the temperature was above gelatinization, the starch exhibited B-type diffraction peaks, with a sharper peak at approximately 20°, indicating an enhanced *V*-type structure. This observation differed from the report of Keeratiburana et al. (2020). They employed amyloglucosidase and maltogenic α-amylase to hydrolyze rice starch; as a result, after the hydrolysis of rice starch, the crystal structure did not undergo any significant changes and retained their A-type crystal structure, which may be attributed to the differences in the raw materials used. Similar to the pullulanase-treated samples, amyloglucosidase also generated short-chain molecules that induced starch chain reorganization, leading to the transformation from an unstable A-type crystal structure to a more stable B-type crystal structure.

As shown in Table 2, native starch had a relative crystallinity of 30.98%. At 50 °C, crystallinity rose with increasing enzyme dosage from 10 U to 100 U. As reported by predecessors, when amylase hydrolyzed starch, it preferentially hydrolyzed the amorphous region of starch that was unstable (Liang et al., 2021). The internal chain segments of starch molecules then rearranged, which enhanced the crystallinity of starch, making the crystal structure perfect and regular. This finding aligned with the results of the *in vitro* digestion experiments with starch, which showed that, with an increase in the amount of enzyme added, the content of RDS decreased, while the content of SDS + RS increased.

**Table 2:** Relative crystallinity, peak intensity ratio and FWHM values of hulless barley starch.

	**RC/%**	**R**_**1045/1022**_	**R**_**1022/995**_	**480 cm**^**−1**^	**855 cm**^**−1**^	**1339 cm**^**−1**^
**Native starch**	30.98 ± 0.53 cd	0.45 ± 0.03e	2.58 ± 0.07a	22.96 ± 0.07a	41.23 ± 0.11b	42.05 ± 0.27a
**A-pullulanase-5**	32.72 ± 0.59c	0.47 ± 0.02e	2.13 ± 0.05b	22.18 ± 0.15a	27.03 ± 0.60e	39.58 ± 1.14b
**A-pullulanase-20**	35.55 ± 1.23b	0.52 ± 0.10d	1.62 ± 0.15d	18.19 ± 0.57c	29.38 ± 0.23c	29.51 ± 0.39d
**A-pullulanase-50**	38.13 ± 0.61a	0.60 ± 0.05 cd	1.11 ± 0.09e	19.23 ± 0.04bc	25.82 ± 0.35f	30.20 ± 1.22d
**B-pullulanase-5**	21.37 ± 1.36 g	1.21 ± 0.11b	0.48 ± 0.04 g	17.18 ± 0.06d	25.79 ± 0.44f	26.29 ± 1.18e
**B-pullulanase-20**	24.66 ± 0.31f	1.90 ± 0.26a	0.37 ± 0.01 h	20.40 ± 0.54b	23.39 ± 0.72 g	23.59 ± 0.99f
**B-pullulanase-50**	17.93 ± 0.45 h	1.94 ± 0.12a	0.35 ± 0.06 h	16.10 ± 0.15e	19.82 ± 0.61 h	21.37 ± 0.47 g
**A-AMG-5**	29.92 ± 0.85d	0.44 ± 0.09e	2.65 ± 0.31a	18.31 ± 0.20c	44.37 ± 1.16a	38.81 ± 1.85b
**A-AMG-20**	32.44 ± 1.73c	0.53 ± 0.04d	2.02 ± 0.24bc	17.46 ± 0.12 cd	43.75 ± 0.82a	34.72 ± 1.23c
**A-AMG-50**	35.85 ± 1.22b	0.77 ± 0.18c	1.89 ± 0.12c	15.33 ± 0.28e	41.46 ± 1.63b	38.39 ± 2.92b
**B-AMG-5**	31.13 ± 0.85 cd	1.14 ± 0.20b	0.82 ± 0.14f	19.23 ± 0.14bc	29.73 ± 0.30c	32.53 ± 1.64 cd
**B-AMG-20**	27.91 ± 0.49e	1.19 ± 0.07b	0.53 ± 0.08 g	20.00 ± 0.86b	28.19 ± 0.26d	22.20 ± 0.53 g
**B-AMG-50**	14.93 ± 0.52i	–	–	20.03 ± 0.97b	23.50 ± 0.37 g	24.97 ± 0.69ef

Data presented as Mean ± SD of three replicate values. Duncan's multiple comparison test (*p* < 0.05) of one-way ANOVA was performed to determine the differences in data. Different letters in the table indicated significant differences in values.

At 60 °C, the relative crystallinity did not increase with an increase in the amount of enzyme added. When the amount of pullulanase added reached 10 U, the relative crystallinity of the B-pullulanase-20 was the highest at 24.66%. When the amount of amyloglucosidase added was 40 U, the relative crystallinity of the B-AMG-5 sample was the highest at 31.13% (Table 2).

The crystallization process of starch is a result of an orderly arrangement of molecules that is influenced by diverse factors, such as temperature, humidity, gelatinization degree and the structure of starch molecules. For instance, amylose shows a higher crystallization ability, while amylopectin shows a weaker crystallization ability. Moreover, directional enzymatic hydrolysis in starch mean that the less compact amorphous region is first hydrolyzed by enzymes; moderate hydrolysis can therefore improve the perfect state of the starch crystals, whereas continuing hydrolysis eventually damages the crystal structure. Notably, when the enzymatic hydrolysis temperature was higher than the gelatinization temperature of the starch samples, the relative crystallinity of starch after enzymatic hydrolysis was lower than that of native starch (Table 2), but the SDS + RS content of the enzymatic starch was significantly higher than that of the natural starch (Table 1). This could be explained by the aforementioned A → B crystal transformation. It has been reported that the enzymatic hydrolysis of type A starch began from the inside of the starch granule and gradually spread outward, which caused the expansion of the surface and internal pores of the granule. Conversely, the enzymatic hydrolysis of type B starch began from the surface of the granule and gradually formed holes or gaps (Sun et al., 2025). The internal holes and channels allowed the starch enzymes to diffuse into the interior of the starch granule during digestion, thereby accelerating the digestion of starch (Ma et al., 2024). Therefore, type B crystals were more stable and more resistant to the action of starch enzymes for decomposition. Meanwhile, the enhanced *V*-type peak at 20° (Fig. 1) signified an increase in amylose-lipid complexes. It was well recognized that retrograded starch (RS3) predominantly adopted a B-type crystalline pattern, whereas amylose-lipid complexes (RS5) were characterized by a V-type crystalline structure. Accordingly, the co-existence of B-type and enhanced V-type crystals in the enzymatically hydrolyzed starch indicated the formation of both RS3 and RS5, leading to an increase in the combined RS3 + RS5 content.

### 3.4 FTIR spectra of starch

**Fig. 2:**
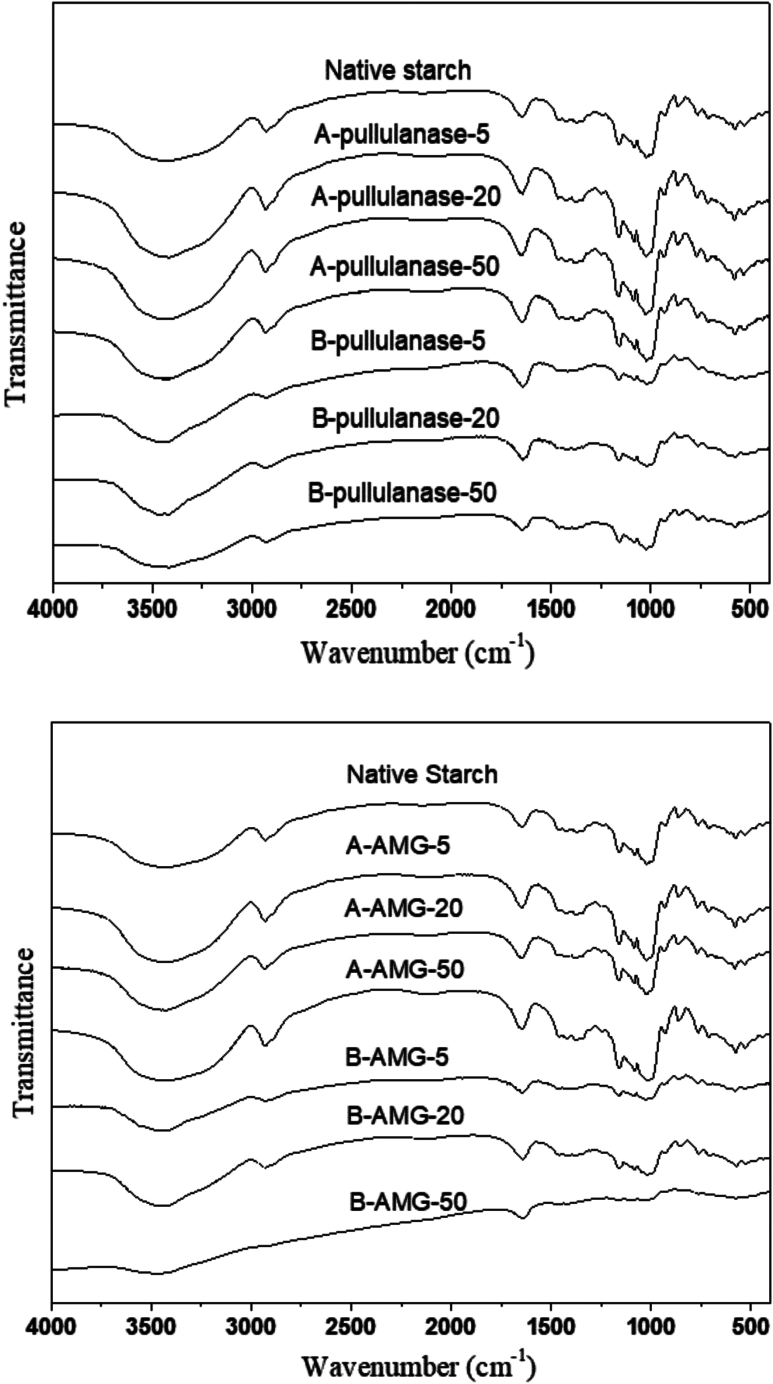
The variation in the FTIR spectrum of starch samples modified by pullulanase and amyloglucosidase under different temperature and concentration conditions.

FTIR spectroscopy was used to assess structural changes. Different functional groups and molecular vibrations generated characteristic peaks, enabling the analysis of starch composition. Fig. 2 displayed the FTIR spectra of native and enzyme-modified starch. Native starch exhibited peaks at approximately 3410, 2930, and 1652 cm^−1^, which corresponded to O—H stretching, -CH₂ stretching, and the amorphous region, respectively. The 1100–1200 cm^−1^ range represented C—C, C—O, and C-O-H vibrations, while those at approximately 930 and 765 cm^−1^ corresponded to C-pyranose C=O and glucopyranose ring vibrations, respectively (Sohrabi et al., 2025).

At 50 °C, the FTIR spectra of treated samples resembled that of native starch, with no new peaks or shifts, indicating no change in chemical bonds—only internal structure rearrangement. Enzymatic treatment increased transmittance at 2930 and 1652 cm^−1^, suggesting molecular disruption and structural rearrangement. The absorption intensities of the characteristic peaks weakened here because of starch molecule destruction during enzymatic treatment (Fig. 2).

At 60 °C, while most peaks remained, some disappeared or changed intensity significantly, suggesting more substantial structural changes. A shift of the 3410 cm^−1^ peak to a higher wavenumber suggested reduced -OH groups and the formation of hydrogen bonds between the molecular chains of the treated starch (Fig. 2).

Peaks at 1045, 1022, and 995 cm^−1^ represented the crystalline structure (ordered glu-cose unit arrangements), amorphous regions (disordered molecular chains), and C-6 hy-droxyl intramolecular hydrogen bonds in starch, respectively. The 1045/1022 cm^−1^ and 1022/995 cm^−1^ ratios (peak intensity ratio) measured the degree of short-range order in starch. The former reflected the relative proportion of crystalline to amorphous regions, where a higher value indicated increased surface crystallinity, while the latter, by com-paring amorphous content to hydrogen-bonded structures, showed that a lower ratio was associated with reduced amorphous content, consistent with enhanced short-range order. Hence, a higher R_1045/1022_ and lower R_1022/995_ reflected increased surface crystallinity and reduced amorphous content. As shown in Table 2, native starch had R_1045/1022_ = 0.45 and R_1022/995_ = 2.58. At 50 °C, both pullulanase and amyloglucosidase increased R_1045/1022_ and decreased R_1022/995_. These trends intensified with higher enzyme concentrations, confirming enhanced molecular ordering. These findings aligned with the observed relative crystallinity of starch. Pullulanase and amyloglucosidase specifically cleaved amylopectin, producing short-chain molecules that reoriented and formed double-helix structures, increasing surface short-range order. Enzymatic hydrolysis typically began in the amorphous regions of starch, which was more susceptible to degradation. This process reduced the R_1022/995_ ratio and enhanced crystallinity. As amylopectin was hydrolyzed, more compact double-helix structures formed, increasing the R_1045/1022_ value and enhancing surface order. This targeted enzymatic hydrolysis of starch has been confirmed in previous studies (Sun et al., 2025). When enzymes interacted with starch, the less stable amorphous regions were hydrolyzed first, leading to a decrease in the R_1022/995_ ratio.

At 60 °C, both enzymes significantly increased R_1045/1022_ and decreased R_1022/995_ (Table 2), indicating a significantly enhanced degree of short-range orderization on the surface of starch granules and a significantly reduced proportion of amorphous regions. At temperatures above the gelatinization point, the original double-helix and crystalline structures were more extensively disrupted. This increased the specific surface area of starch granules, facilitating enzyme access (Zhang et al, 2025b). Consequently, more short-chain molecules were produced, which reorganized into densely packed helices and more regular crystal structures, generating complex granular architectures. Studies have shown that early hydrolysis rapidly degraded amorphous regions, enhancing overall crystalline stability (Sun et al., 2025). XRD analysis further supported these findings. During starch retrogradation, unstable A-type crystals gradually transformed into more stable B-type forms, with an increased proportion of *V*-type crystals. These structural transformations corresponded with *in vitro* digestion results: when enzymatic hydrolysis occurred above gelatinization temperature, SDS + RS content increased significantly, reflecting both the increased surface order and the transformation of amorphous regions.

### 3.5 Raman spectra of starch

**Fig. 3:**
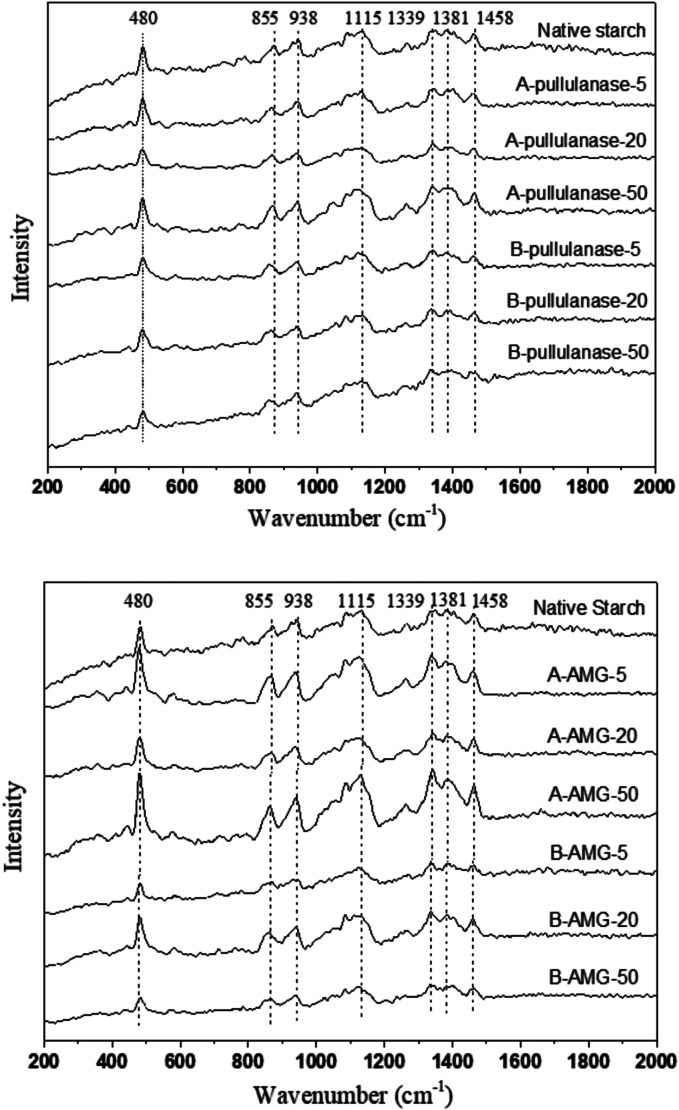
The variation in the Raman spectrum of starch samples modified by pullulanase and amylogluco-sidase under different temperature and concentration conditions.

Fig. 3 presented the Raman spectra of starch samples. Native starch displayed characteristic peaks at approximately 480, 855, 938, 1115, 1339, 1381, and 1458 cm^−1^. Post-enzyme treatment, the modified starch exhibited similar peak positions and shapes, indicating no formation of new chemical bonds—only internal structural rearrangement. However, variations in peak intensities suggested differences in molecular structure compared to the original starch.

The peaks at 480, 855, and 1339 cm^−1^ were associated with short-range order and the relative content of double- and single-helical structures in starch. A larger Full-Width at Half Maximum (FWHM) at these peaks indicated lower order and fewer single/double helices. As shown in Table 2, native starch had FWHM values of 22.96, 41.23, and 42.05 at these respective peaks. In A-pullulanase-treated samples, the FWHM at 480 cm^−1^ decreased, indicating enhanced surface short-range order. Similarly, reductions in FWHM at 855 and 1339 cm^−1^ suggested increased formation of amylose and amylopectin helices. At 50 °C, amyloglucosidase treatment also reduced the FWHM at 480 cm^−1^, further increasing surface order. However, FWHM at 855 cm^−1^ was higher than in native starch, likely due to the enzyme hydrolyzing both amorphous and crystalline regions. This dual-region hydrolysis has been observed in previous studies. Zhang et al. (2006) found that porcine pancreatic α-amylase and amyloglucosidase hydrolyzed both crystalline and amorphous regions of corn starch without significantly altering overall crystallinity during different hydrolysis stages. Meanwhile, FWHM values at 1339 cm^−1^ for A-AMG samples slightly decreased, indicating an increase in single-helix structures.

At 60 °C, pullulanase and amyloglucosidase treatments resulted in notable FWHM reductions at 480, 855, and 1339 cm^−1^, signifying enhanced surface short-range order and more helix formation. According to the structural model of retrograded starch proposed by Edwards et al. (2014), some amorphous regions became incorporated into crystalline zones during reorganization, while others remained on the surface. These embedded amorphous regions were partially shielded during digestion, resulting in a more compact granular structure in modified starch compared to the original form. This interpretation was consistent with data from *in vitro* digestion, XRD, and FTIR analyses. XRD showed a transformation from A-type to B-type crystal structures, which were more resistant to enzymatic hydrolysis, along with an increase in *V*-type crystal structures formed by single-helix crystallization. FTIR results showed a higher R_1045/1022_ ratio and a lower R_1022/995_ ratio, indicating an increased proportion of crystalline regions and reduced amorphous content. Therefore, enzymatic modification at temperatures above starch gelatinization significantly reduced the RDS content while significantly increasing the SDS + RS content, driven by enhanced short-range order and structural reorganization of starch granules.

### 3.6 Particle size and diameter distribution of starch

**Fig. 4:**
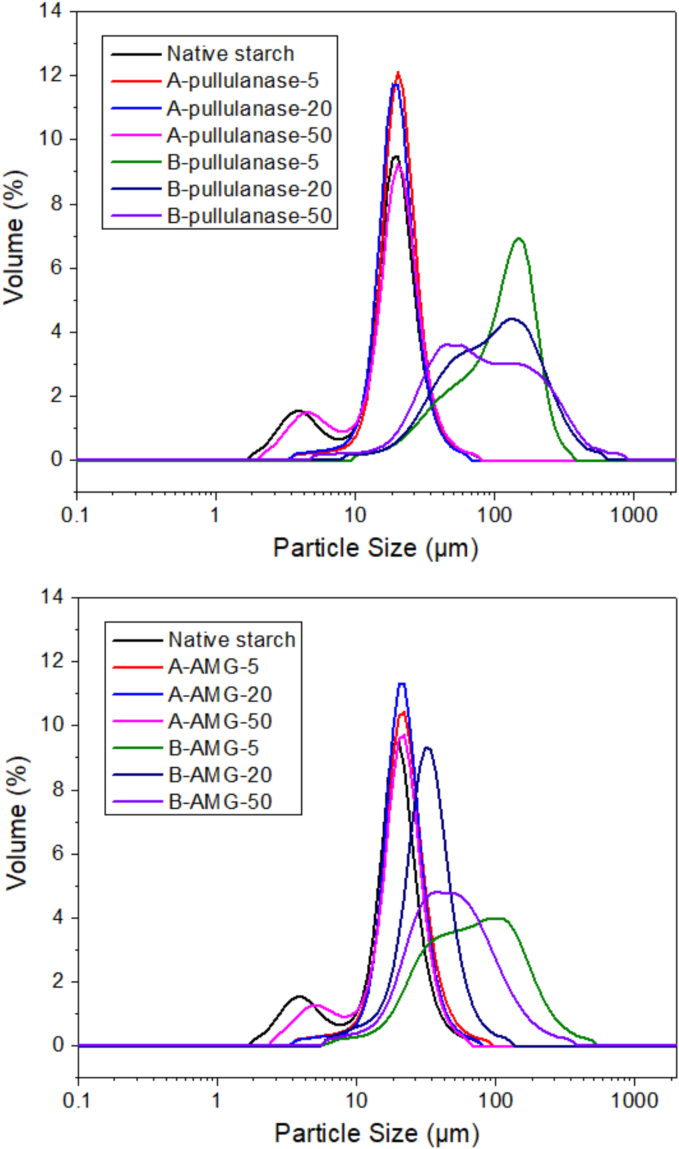
The variation in the particle size distribution of starch samples modified by pullulanase and am-yloglucosidase under different temperature and concentration conditions.

Fig. 4 showed the particle size distribution of starch samples. Native starch displayed two distinct peaks. As listed in Table 3, Peak 1 had a peak particle size of 3.57 μm (range: 1.64–7.12 μm), accounting for approximately 15% of the total distribution. Peak 2 had a peak particle size of 18.86 μm (range: 7.78–74.86 μm), accounting for approximately 85% of the total distribution. Here, the peak particle size refers to the mode of each sub-population, *i.e.*, the particle diameter at the maximum of the volume distribution peak.

**Table 3:** Particle size of hulless barley starch.

	**Peak 1**	**Peak 2**	**D**_**50**_
**Native starch**	3.57 ± 0.03a	18.86 ± 0.07 h	17.62 ± 0.03 h
**A-pullulanase-5**	–	19.49 ± 0.12gh	19.49 ± 0.12gh
**A-pullulanase-20**	–	18.33 ± 0.23 h	18.33 ± 0.23 h
**A-pullulanase-50**	4.16 ± 0.02a	19.49 ± 0.03 gh	18.30 ± 0.04 h
**B-pullulanase-5**	–	105.9 ± 3.78a	105.9 ± 3.78a
**B-pullulanase-20**	–	90.08 ± 4.47b	90.08 ± 4.47b
**B-pullulanase-50**	–	75.27 ± 0.85c	75.27 ± 0.85c
**A-AMG-5**	–	20.62 ± 0.05 g	20.62 ± 0.05 g
**A-AMG-20**	–	20.35 ± 0.03 g	20.35 ± 0.03 g
**A-AMG-50**	4.42 ± 0.03a	20.24 ± 0.09 g	19.30 ± 0.08 gh
**B-AMG-5**	–	63.38 ± 2.54d	63.38 ± 2.54d
**B-AMG-20**	–	30.28 ± 0.32f	30.28 ± 0.32f
**B-AMG-50**	–	46.21 ± 2.16e	46.21 ± 2.16e

Data presented as Mean ± SD of three replicate values. Duncan's multiple comparison test (*p* < 0.05) of one-way ANOVA was performed to determine the differences in data. Different letters in the table indicated significant differences in values.

At 50 °C, enzyme-modified hulless barley starch exhibited a single distribution peak. The peak particle sizes for A-pullulanase-5, A-pullulanase-20, A-AMG-5, and A-AMG-20 were 19.49, 18.33, 20.62, and 20.35 μm, respectively (Table 3). This suggested that smaller starch particles, with their larger surface area, are more susceptible to enzymatic hydrolysis. While pullulanase-modified starch samples showed no significant size difference compared with the original starch, amyloglucosidase-modified samples had significantly larger peak particle sizes. Notably, A-pullulanase-50 and A-AMG-50 samples still exhibited two distribution peaks.

At 60 °C, enzyme modification resulted in a single broad peak in the size distribution. Pullulanase-modified starch had particle sizes ranging from 75.27 to 105.9 μm, and amyloglucosidase-modified starch had particle sizes ranging from 46.21 to 63.38 μm (Table 3). They were both much larger than native starch. The D_50_ value (median particle size) of native starch was 17.62 μm, which was derived from the cumulative volume distribution of the entire sample. After modification at 50 °C, D_50_ values ranged from 18.30 to 19.49 μm for pullulanase-treated samples and from 19.30 to 20.62 μm for amyloglucosidase-treated samples (Table 3). When the temperature was higher than the gelatinization temperature (at 60 °C), the D50 values increased significantly, aligning with the broader particle size distributions (from 75.27 to 105.9 μm for pullulanase-treated samples and from 46.21 to 63.38 μm for amyloglucosidase-treated samples).

### 3.7 SEM results of starch

**Fig. 5:**
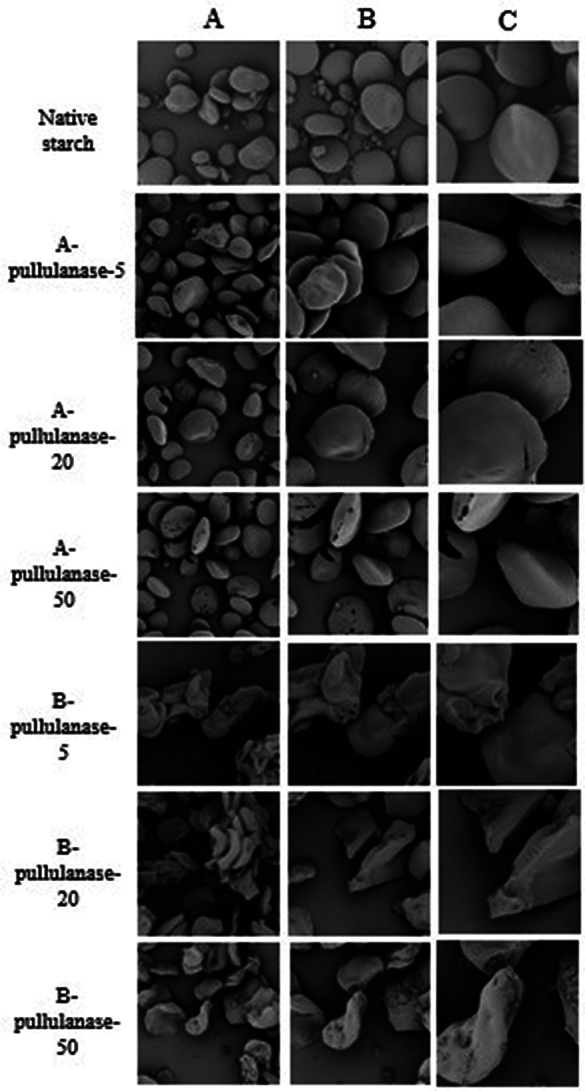
The SEM pictures of starch samples modified by pullulanase under different temperature and concentration conditions. (A: **×**3000; B: **×**5000; C: **×**10,000).

Fig. 5 presented SEM images of native and pullulanase-modified samples. Native starch granules were flat, elliptical, and smooth. At 50 °C (below gelatinization temperature), pullulanase-modified starch regained its general shape but showed surface erosion-cracks and pores appeared due to enzymatic action. As the pullulanase concentration increased, erosion intensified, and the cracks and voids on the surface increased in size and number. At 60 °C, the morphology of the pullulanase-modified starch granules changed significantly. The elevated temperature likely caused granule swelling, disrupting the structure of the starch granules.

**Fig. 6:**
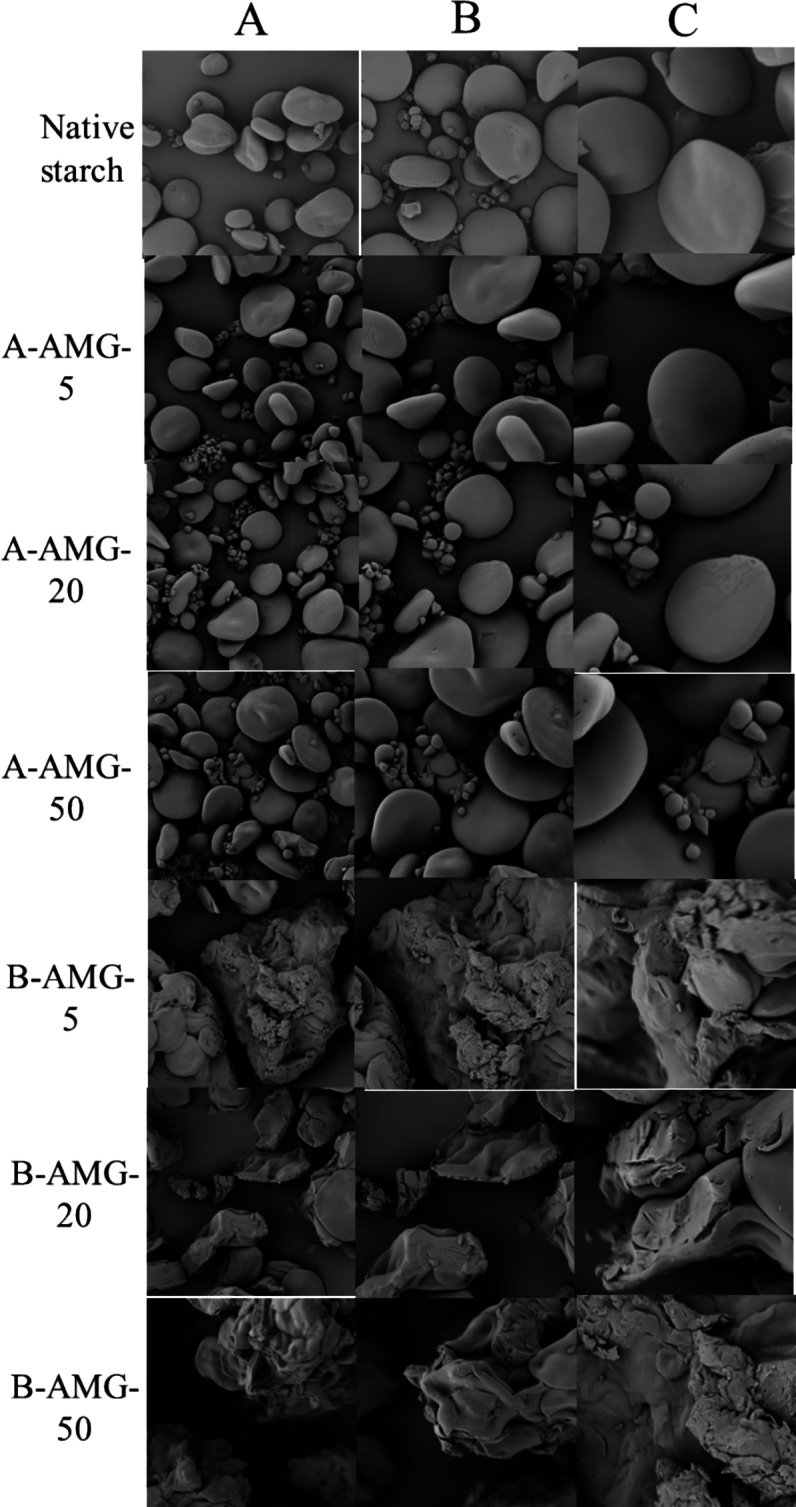
The SEM pictures of starch samples modified by amyloglucosidase under different temperature and concentration conditions. (A: ×3000; B: ×5000; C: ×10,000).

Fig. 6 presented SEM images of native starch and amyloglucosidase-modified samples. At 50 °C, which was below the gelatinization temperature, amyloglucosidase caused only minor morphological changes, and the granules retained their overall shape but displayed slightly roughened surfaces. This limited alteration was attributable to the compact, semi-crystalline architecture of native starch granules, which restricted the accessibility of amyloglucosidase. At 60 °C, however, a drastically different morphology was observed. The elevated temperature was above the onset of gelatinization, causing the granules to swell, absorb water and lose their crystalline order. This structural loosening allowed amyloglucosidase to penetrate the granule interior and extensively hydrolyze the starch chains. Consequently, the granules underwent severe disruption, with marked surface collapse, fusion and loss of individual particle definition.

The structural breakdown of the granules by amyloglucosidase was considerably more extensive than that observed with pullulanase at 60 °C. This difference could be explained by their distinct action modes: pullulanase specifically hydrolyzed α-1,6 branch points and produced linear chains without rapidly degrading them, which partially preserved the granular framework; in contrast, amyloglucosidase progressively broke down the entire starch molecule into glucose, leading to a more comprehensive collapse of the swollen granular structure and a more thoroughly disintegrated morphology.

### 3.8 Thermal and pasting properties

**Fig. 7:**
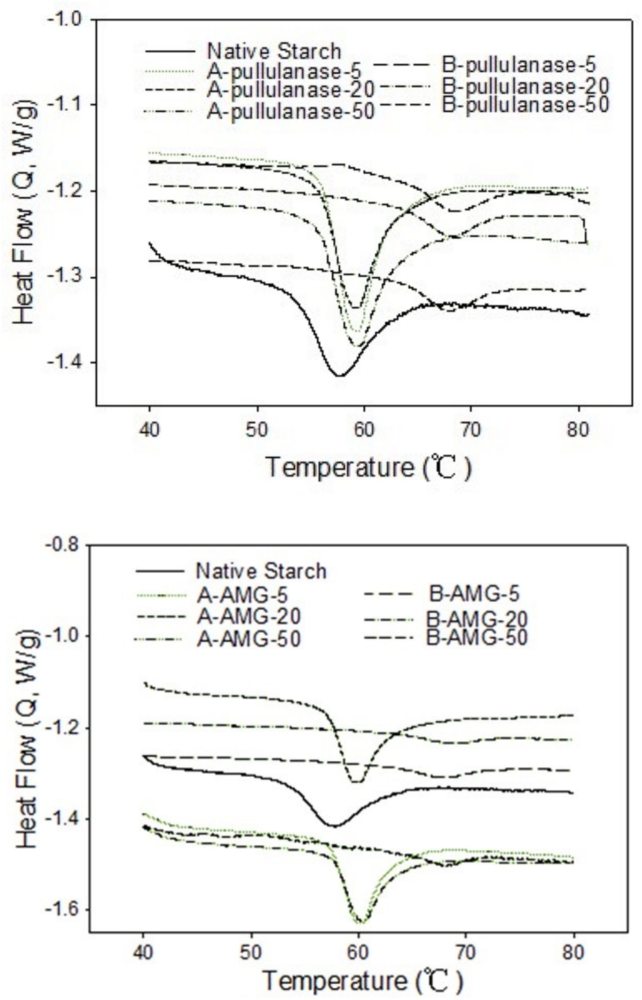
Gelatinization thermograms of starch samples modified by pullulanase and amyloglucosidase under different temperature and concentration conditions.

Fig. 7 showed DSC heat flow diagrams of starch samples. Table 4 summarized the relevant thermodynamic parameters of the samples. For native starch, the *To*, *Tp*, *Tc*, *Tc-To* and *ΔH* values were 53.71 °C, 57.80 °C, 62.29 °C, 8.58 °C and 5.56 J/g, respectively.

**Table 4:** The thermal properties of hulless barley starch.

	***To***	***Tp***	***Tc***	***Tc*-*To***	***ΔH***
**Native starch**	53.71 ± 0.25e	57.80 ± 0.04c	62.29 ± 0.12b	8.58 ± 0.07a	5.56 ± 0.34d
**A-pullulanase-5**	56.95 ± 0.11 cd	59.69 ± 0.55b	62.17 ± 0.44b	5.22 ± 0.28d	11.7 ± 1.83a
**A-pullulanase-20**	55.99 ± 0.84d	59.49 ± 0.32b	62.32 ± 0.27b	6.33 ± 0.46c	9.59 ± 1.81b
**A-pullulanase-50**	55.41 ± 0.95d	59.77 ± 0.57b	62.96 ± 0.04b	7.55 ± 0.73b	9.47 ± 0.27b
**B-pullulanase-5**	63.89 ± 0.44ab	68.46 ± 0.92a	72.60 ± 0.26a	8.71 ± 0.65a	2.07 ± 0.86ef
**B-pullulanase-20**	63.67 ± 0.48ab	68.20 ± 0.06a	72.00 ± 0.04a	8.33 ± 0.23a	2.27 ± 0.01ef
**B-pullulanase-50**	63.21 ± 0.34b	67.81 ± 0.01a	71.98 ± 0.14a	8.77 ± 0.15a	2.76 ± 0.16e
**A-AMG-5**	57.22 ± 0.49c	60.01 ± 0.06b	62.83 ± 0.28b	5.61 ± 0.31 cd	8.37 ± 0.16c
**A-AMG-20**	57.89 ± 0.55c	59.97 ± 0.14b	62.43 ± 0.30b	4.54 ± 0.29e	8.54 ± 0.45bc
**A-AMG-50**	57.19 ± 0.95c	60.10 ± 0.4b	62.57 ± 0.55b	5.38 ± 0.67d	7.58 ± 0.78c
**B-AMG-5**	63.50 ± 0.17b	68.23 ± 0.43a	71.87 ± 0.27a	8.37 ± 0.38a	1.66 ± 0.66ef
**B-AMG-20**	64.28 ± 0.54a	67.85 ± 0.93a	72.35 ± 0.07a	8.07 ± 0.42ab	1.89 ± 0.2ef
**B-AMG-50**	64.85 ± 0.77a	68.18 ± 0.07a	72.54 ± 0.15a	7.69 ± 0.30b	1.53 ± 0.27f

Data presented as Mean ± SD of three replicate values. Duncan's multiple comparison test (*p* < 0.05) of one-way ANOVA was performed to determine the differences in data. Different letters in the table indicated significant differences in values.

At 50 °C, the *To* value for pullulanase-modified starch ranged from 55.41 °C to 56.95 °C, the *Tp* value ranged from 59.69 °C to 59.77 °C, and the *Tc* value ranged from 62.17 °C to 62.32 °C. The *To* and *Tp* values for pullulanase-modified starch were significantly higher than those for the native samples, indicating that the thermal stability of the samples increased. Additionally, the *Tc-To* ranges for the samples treated with A-pullulanase-5, A-pullulanase-20, and A-pullulanase-50 were 5.22 °C, 6.33 °C, and 7.55 °C, respectively, which were notably lower than those for the original starch granules. The respective *ΔH* values were 11.7, 9.59, and 9.47 J/g, which were significantly higher than those for the native starch. This suggested that the energy required to disrupt the double-helix structure and crystalline order of the starch samples increased.

At 60 °C, the *To* value for amyloglucosidase-modified starch ranged from 57.19 °C to 57.89 °C, the *Tp* value ranged from 59.97 °C to 60.10 °C, the *Tc* value ranged from 62.43 °C to 62.82 °C, the *Tc-To* ranges were 4.54 °C–5.61 °C, and the *ΔH* values ranged from 7.58 to 8.54 J/g. Of them, the *To*, *Tp*, and *ΔH* values were significantly higher than those for native starch, and the *Tc*-*To* value was notably lower than that of native starch.

Overall, when the enzymatic hydrolysis temperature was lower than the gelatinization temperature of starch, the enzyme-modified starch and the native samples had significantly different thermodynamic properties, likely due to enzyme modification-induced structural changes in starch. Starch gelatinization appeared as an endothermic peak in the DSC spectrum because of the melting of starch crystals. This melting implied that the ordered structure of starch chains was transformed into a disordered one (Geng et al., 2025). The amorphous region of starch played a crucial role in crystal melting. Compared with the starch crystals, the structure of the amorphous region was looser, which allowed small molecules to diffuse more easily within it (Ai & Jane, 2018). In this work, enzymatic hydrolysis at lower temperatures increased the proportion of crystalline regions, whereas decreased the proportion of amorphous regions in starch, thereby improving its thermal stability.

Table 4 also presented the *To*, *Tp*, *Tc*, *ΔH*, and *Tc*-*To* values for the pullulanase-modified starch samples at 60 °C. Among them, the *To*, *Tp*, and *Tc* values were significantly higher than those for the native starch samples, the *ΔH* value was significantly lower than that for the original starch granules, and little difference was noted in the *Tc*-*To* values. Similarly, the *To*, *Tp*, and *Tc* values for the amyloglucosidase-modified starch samples were significantly higher than those for the native starch samples, and the *ΔH* value was significantly lower than that for the original starch granules. The results indicated that the thermal stability of the enzyme-treated samples improved. However, the energy required to disrupt the double-helix structure and crystalline order of the samples decreased, likely because enzyme treatment induced structural changes in starch. On the one hand, structural characterizations by XRD, FTIR, and Raman proved that the amorphous region of the modified starch was first rapidly hydrolyzed in the early stage of enzyme action, thus increasing the stability of starch granules during gelatinization. However, as enzymatic hydrolysis continued, both amorphous and crystalline regions of starch were degraded, thereby decreasing the energy required to disrupt the double-helix structure and crystalline order. On the other hand, the crystal structure type of hulless barley starch changed after enzymatic hydrolysis and retrogradation. Starches with different crystal structure types had varying thermodynamic properties. Lan et al. (2015) found *ΔH* values in that order: A-type > C-type > B-type. The aforementioned results indicated that the crystal structure of starch is closely related to its thermal properties, which were influenced by the double-helix structures formed by different starch chains.

### 3.9 Rheological properties of starch

#### 3.9.1 Steady-state shear characteristics

The rheological properties of starch samples were analyzed using the power-law model. As shown in Table 5, the model exhibited a strong fit, with determination coefficients (R^2^) ranging from 0.91 to 0.99 across all samples. During the shear-increasing stage, the consistency coefficient (k) and flow behavior index (n) for native starch were 3.8342 Pa·s^n^ and 0.1253, respectively, with an R^2^ of 0.9387. In the shear-decreasing stage, the k and n values for native starch were 1.5527 Pa·sⁿ and 0.7325, respectively, with an R^2^ of 0.9899. Since the flow behavior index *n* < 1, hulless barley starch paste exhibited non-Newtonian pseudoplastic behavior-characterized by shear thinning, where the viscosity decreased with the increasing shear rate. This phenomenon was typically attributed to molecular alignment during flow, deformation of swollen molecules during flow, and partial disintegration of the gel due to the disruption of hydrogen and other secondary bonds.

**Table 5:** The power law parameters of hulless barley starch.

**Parameters**	**Upward**			**Downward**		
	**k**	**n**	**R**^**2**^	**k**	**n**	**R**^**2**^
**Native starch**	3.8342 ± 0.42a	0.1253 ± 0.04f	0.9387	1.5527 ± 0.31a	0.7325 ± 0.05c	0.9899
**A-pullulanase-5**	3.4186 ± 0.74a	0.1277 ± 0.07f	0.9265	1.4364 ± 0.27a	0.7602 ± 0.01bc	0.9741
**A-pullulanase-20**	2.6516 ± 0.12b	0.1582 ± 0.03e	0.9813	1.3433 ± 0.22ab	0.8182 ± 0.03b	0.9838
**A-pullulanase-50**	2.2379 ± 0.29bc	0.1639 ± 0.01e	0.9350	1.2514 ± 0.18b	0.8506 ± 0.08ab	0.9937
**B-pullulanase-5**	0.6914 ± 0.11de	0.2051 ± 0.07d	0.9433	0.08622 ± 0.03e	0.9053 ± 0.13a	0.9653
**B-pullulanase-20**	0.5920 ± 0.07de	0.4921 ± 0.03b	0.9273	0.07294 ± 0.01e	0.9104 ± 0.11a	0.9619
**B-pullulanase-50**	0.2804 ± 0.06e	0.7458 ± 0.12a	0.9785	0.03004 ± 0.01e	1.0765 ± 0.16a	0.9931
**A-AMG-5**	2.7593 ± 0.39b	0.2189 ± 0.09d	0.9428	1.0372 ± 0.18c	0.5510 ± 0.07d	0.9816
**A-AMG-20**	1.9031 ± 0.23c	0.2821 ± 0.04c	0.9517	0.9371 ± 0.14c	0.5792 ± 0.05d	0.9739
**A-AMG-50**	1.1214 ± 0.28d	0.3388 ± 0.15c	0.9100	0.6188 ± 0.09 cd	0.6497 ± 0.12 cd	0.9966
**B-AMG-5**	0.9797 ± 0.08d	0.2024 ± 0.06d	0.9265	0.4859 ± 0.05d	0.6836 ± 0.06c	0.9977
**B-AMG-20**	0.5257 ± 0.03de	0.2072 ± 0.03d	0.9872	0.4367 ± 0.06d	0.7065 ± 0.14c	0.9941
**B-AMG-50**	0.4920 ± 0.05e	0.2376 ± 0.04d	0.9318	0.4037 ± 0.03d	0.8310 ± 0.11ab	0.9827

Data presented as Mean ± SD of three replicate values. Duncan's multiple comparison test (*p* < 0.05) of one-way ANOVA was performed to determine the differences in data. Different letters in the table indicated significant differences in values.

At 50 °C, pullulanase-modified starch samples showed k values between 2.2379 and 3.4185 Pa·sⁿ and n values from 0.1277 to 0.1639 in the shear-increasing stage. During the shear-decreasing stage, the k values ranged from 1.2514 to 1.4364 Pa·sⁿ and n values from 0.7602 to 0.8506. At 60 °C, the k values for the pullulanase-modified starch samples dropped to 0.2804–0.6914 Pa·sⁿ, whereas the n values increased to 0.2051–0.7458 during the shear-increasing stage. During the shear-decreasing stage, the k values dropped further to 0.03004–0.08622 Pa·sⁿ, with n values ranging from 0.9053 to 1.0765. With increasing pullulanase concentration, the k values continuously decreased, whereas n values increased, indicating reduced thickening ability and weaker shear thinning behavior.

A similar trend was observed in starch samples modified by amyloglucosidase, suggesting both enzymes reduced the shear viscosity of hulless barley starch. This reduction likely resulted from enzymatic hydrolysis of amylopectin into more short-chain molecules, increasing the relative amylose content in the starch system. During gelatinization, amylose more readily leached from granules, forming a weak matrix that encapsulates residual granules (Wang et al., 2026; Xu et al., 2026). This structure was more susceptible to breakdown, thereby reducing the k value. Additionally, higher enzyme concentrations may lead to further amylose degradation and structural damage, contributing to a continued decline in k values (Xu et al., 2026).

#### 3.9.2 Dynamic rheological characteristics

The storage modulus (G') and loss modulus (G") reflected the elastic and viscous components of starch, respectively, while the tan δ value, derived from the G"/G' ratio, indicated the viscoelastic nature of starch samples. As shown in Table 6, enzyme modification increased the temperature (T_G′max_) at which the storage modulus (G′) reached its maximum, likely due to the breakdown of amylopectin into shorter chains that reoriented, entangled, and formed more compact double-helix structures.

**Table 6:** The dynamic rheological properties of hulless barley starch.

**Parameters**	**Heating**					**Cooling**	
	T_*G′*max_	*G'*_max_	tan *δ*_*G′*max_	*G′*_90°C_	tan *δ*_90°C_	*G′*_25°C_	tan *δ*_25°C_
**Native starch**	67.53 ± 0.29e	2673 ± 26.48ef	0.2456 ± 0.07b	801.3 ± 8.92f	0.2019 ± 0.05a	2217 ± 25.32e	0.07234 ± 0.003a
**A-pullulanase-5**	67.49 ± 0.13e	2714 ± 38.39e	0.2331 ± 0.11c	899.7 ± 3.29f	0.1884 ± 0.03b	2592 ± 47.31d	0.06828 ± 0.007a
**A-pullulanase-20**	69.33 ± 0.82d	2992 ± 27.47d	0.2219 ± 0.06d	932.5 ± 11.48e	0.1851 ± 0.06b	2634 ± 38.92d	0.06743 ± 0.018a
**A-pullulanase-50**	70.52 ± 1.29c	3044 ± 29.58d	0.1839 ± 0.09e	1013 ± 15.29d	0.1788 ± 0.02b	2791 ± 21.33c	0.05911 ± 0.001b
**B-pullulanase-5**	70.91 ± 1.17bc	3910 ± 35.69a	0.1512 ± 0.13 g	1149 ± 12.54c	0.1283 ± 0.07d	3018 ± 56.72b	0.04276 ± 0.001 cd
**B-pullulanase-20**	71.45 ± 1.64b	3729 ± 23.84b	0.1663 ± 0.17f	1085 ± 9.42d	0.1057 ± 0.04e	2971 ± 39.09b	0.04193 ± 0.012 cd
**B-pullulanase-50**	72.17 ± 1.81a	3639 ± 35.47b	0.1588 ± 0.01 g	1046 ± 16.77d	0.1021 ± 0.09e	2824 ± 28.55c	0.04722 ± 0.006c
**A-AMG-5**	67.82 ± 0.99e	2612 ± 19.88f	0.2587 ± 0.02a	747.6 ± 7.32 g	0.1548 ± 0.06c	2793 ± 21.78c	0.04419 ± 0.003c
**A-AMG-20**	68.37 ± 0.62e	2704 ± 25.74e	0.2555 ± 0.15a	824.1 ± 2.13f	0.1511 ± 0.08c	2602 ± 33.56d	0.05001 ± 0.009bc
**A-AMG-50**	68.81 ± 0.84de	2798 ± 17.47e	0.2516 ± 0.07a	856.9 ± 13.91f	0.1482 ± 0.02c	2588 ± 14.95d	0.05838 ± 0.010b
**B-AMG-5**	70.19 ± 0.33c	3301 ± 16.47c	0.2078 ± 0.12d	1385 ± 17.68a	0.1297 ± 0.03d	3295 ± 32.36a	0.03698 ± 0.007d
**B-AMG-20**	69.23 ± 0.75d	3378 ± 36.27c	0.1674 ± 0.15f	1224 ± 12.74b	0.1152 ± 0.05e	3217 ± 35.74a	0.03917 ± 0.002 cd
**B-AMG-50**	69.72 ± 0.80d	3365 ± 27.48c	0.1632 ± 0.08f	1297 ± 15.53ab	0.1123 ± 0.02e	3058 ± 29.85b	0.04010 ± 0.005 cd

Data presented as Mean ± SD of three replicate values. Duncan's multiple comparison test (*p* < 0.05) of one-way ANOVA was performed to determine the differences in data. Different letters in the table indicated significant differences in values.

For native starch, G'max, G′90 °C and G′25 °C were 2673, 801.3, and 2217, respectively, while corresponding tan δ_G′max_, tan δ_90°C_ and tan δ_25°C_ values were 0.2456, 0.2019, and 0.07234. Generally, tanδ >1 indicated viscous behavior, 0.1 < tanδ <1 suggestd weak gel characteristics and tanδ <0.1 implied strong gel characteristics. Compared with native starch, samples modified by pullulanase/amyloglucosidase had an increased G' and a decreased tan δ value, indicating enhanced elasticity and reduced viscosity. This behavior could be attributed to the conversion of amylopectin into shorter chains, which increased amylose content and encouraged structural rearrangement. Starch viscosity mainly depended on the amylose content, the branch length distribution of amylopectin, and the presence of certain functional groups and lipids in starch. Amylose inhibited granule swelling, whereas long-branched amylopectin promoted it, leading to increased viscosity during heating (Wang et al., 2025). Therefore, enzymatic modification of amylose and amylopectin within starch granules significantly altered the viscosity characteristics during starch gelatinization.

These findings aligned with previous studies. Li et al. (2017) reported that debranching enzyme treatment reduced peak viscosity by 56% in potato starch and 15% in corn starch. Similarly, Li, Li and Guo. (2020a) observed a lower peak viscosity in pullulanase-modified wheat starch, attributed to longer amylose chains that inhibit swelling. Furthermore, modifications using α-amylase and glucoamylase introduced porous structures in granules, with larger pore sizes and higher pore area ratios correlating negatively with peak viscosity.

## 4 Conclusion

In this study, pullulanase and amyloglucosidase modifications effectively reduced RDS while increasing SDS or RS in hulless barley starch. With increased enzyme dosage, RDS decreased progressively. Enzymatic hydrolysis above starch gelatinization temperature enhanced anti-digestibility more significantly. After enzymatic degradation, the starch crystal structure transformed from unstable A-type to stable B-type, accompanied by altered relative crystallinity, and increased short-range ordered and helical structures. Enzymatic hydrolysis broke down amylopectin into shorter chains, increasing amylose content, and facilitated molecular reorientation, entanglement, and arrangement to form more compact double helices and perfect crystalline structures. These findings provide valuable theoretical and technical support for advanced processing and high-value utilization of hulless barley starch.

## CRediT authorship contribution statement

**Xinzhe Gu:** Writing – review & editing, Writing – original draft. **Zufei Cao:** Formal analysis, Data curation. **Yu Wang:** Methodology, Investigation. **Mingyuan Zheng:** Writing – review & editing. **Xiaohui Liu:** Supervision, Resources, Project administration. **Wei Zhang:** Resources, Conceptualization.

## Declaration of competing interest

The authors declare that they have no known competing financial interests or personal relationships that could have appeared to influence the work reported in this paper.

## Data Availability

Data will be made available on request.
